# A Roadmap for Global Acceleration of Genomics Integration Across Nursing

**DOI:** 10.1111/jnu.12552

**Published:** 2020-04-17

**Authors:** Emma Tonkin, Kathleen A. Calzone, Laurie Badzek, Caroline Benjamin, Anna Middleton, Christine Patch, Maggie Kirk

**Affiliations:** ^1^ Associate Professor of Genomic Healthcare University of South Wales Pontypridd Wales UK; ^2^ *Xi*, Research Geneticist, National Institutes of Health National Cancer Institute, Center for Cancer Research, Genetics Branch Bethesda MD USA; ^3^ *Alpha Rho, Nu Omega*, Dean and Professor, Penn State College of Nursing Pennsylvania State University University Park PA USA; ^4^ Genetic Counsellor Liverpool Women’s NHS Hospital Trust Liverpool England UK; ^5^ Head of Society and Ethics Research, Connecting Science, Wellcome Genome Campus; Professor, Faculty of Education University of Cambridge Cambridgeshire UK; ^6^ Clinical Lead for Genetic Counselling, Genomics England, London, UK; Principal Staff Scientist, Society and Ethics Research, Connecting Science, Wellcome Genome Campus, Cambridge, Cambridgeshire, UK; Visiting Professor Sheffield Hallam University Sheffield South Yorkshire UK; ^7^ Senior Author, Emeritus Professor of Genetics Education University of South Wales Pontypridd Rhondda Cynon Taff UK

**Keywords:** Implementation, genomics, leadership, nursing, roadmap, strategy

## Abstract

**Purpose:**

The changes needed to accelerate integration of genomics across nursing are complex, with significant challenges faced globally. Common themes lend themselves to a coordinated and collaborative strategic approach to sustained change. We aim to synthesize the outputs of a research program to present a roadmap for nursing leadership to guide integration of genomics across practice.

**Design:**

Mixed methods involving a purposive sample of global nursing leaders and nursing organizations in a sustained, highly interactive program.

**Methods:**

Experts in nursing, health care and healthcare services, policy, and leadership were recruited. Online surveys preceded a 3‐day residential meeting utilizing participatory methods and techniques to gain consensus on the essential elements of a roadmap to promote genomics integration.

**Findings:**

Twenty‐three leaders representing 19 countries and seven organizations participated overall. Data on the scope and status of nursing, genomics health care, and resources have been synthesized. Participants identified 117 facilitators to genomics integration across diverse sources. Barriers and priorities identified were mapped to the constructs of the Consolidated Framework for Implementation Research. The roadmap is underpinned by a maturity matrix created by participants to guide and benchmark progress in genomics integration.

**Conclusions:**

Nurse leaders seeking to accelerate change can access practical guidance with the roadmap, underpinned by support through the Global Genomics Nursing Alliance and its strategic priorities.

**Clinical Relevance:**

Genomics is shaping the future of healthcare, but change is needed for integration across nursing. This practical roadmap, adaptable to local health systems and clinical and educational contexts, is relevant to nurse leaders aiming to accelerate change.

The Global Genomics Nursing Alliance (G2NA, www.g2na.org) was established in 2017 to support nurses to realize their full potential in integrating genomics across nursing practice, to improve healthcare for all. Built on a collaborative network, it aims to provide guidance and practical support for nurses globally. It was created in response to the acknowledged widespread deficits in nurses’ knowledge and understanding of genomics (Calzone et al., 2018, 2018a), also recognizing its increasing importance across nursing (Camak, 2016) and that genomics itself is set to transform the delivery of care for patients (Stark et al., 2019). In this article, as part of our efforts to support transformational change in genomics and healthcare delivery, we aim to present a plan of action (roadmap) for nurse leaders to guide and benchmark strategic developments to integrate genomics across nursing.

## Background

### Genomics and Nursing

Technological advances have brought new opportunities for clinical application of genomics. Braithwaite et al. (2018) identify it as one of five core trends shaping the future of healthcare. However, Turnbull et al. (2018) warned that rapid expansion of genomics within clinical services requires concomitant changes to infrastructure alongside education, upskilling the healthcare workforce and public engagement. They cite the U.K. 100,000 Genomes Project as an exemplar of the application of whole genome sequencing catalyzing developments across the National Health Service. Stark et al. (2019) noted that at least 14 countries have invested a combined total of more than $4 billion to establish national genomic medicine initiatives. They contend that accelerating implementation of genomic health care is a global responsibility to enable health benefits for all.

Nurses are essential to realizing these benefits and to contribute to changes in health systems (Tonkin et al., submitted). The sheer size of the global workforce and the diversity of the professional role and settings in which nurses practice make them ideally placed to deliver genomic health care, from novice to advanced practice levels, which may include diagnostic and prescriptive responsibilities. However, although well positioned to take a lead in implementing genomic innovations, nurses are not well prepared to do so (Williams, Feero, Leonard, & Coleman, 2017). A number of studies have addressed approaches to effective, sustainable implementation, but to the best of our knowledge there have been no international collaborative efforts to accelerate genomics integration across nursing.

### Nurse Leadership

The World Health Organization (WHO) sees nurses and midwives as essential to its achievement for universal health coverage. Its Nursing Now! campaign, run in collaboration with the International Council of Nurses, seeks to empower nurses and midwives to play a core role in tackling 21st century health challenges (www.nursingnow.org). Part of its focus is on ensuring that nurses and midwives have a more prominent voice in health policymaking, and that more nurses are recruited into leadership positions. Instead, Salvage and White (2019, p. 148) noted that nurses “carry out the policy decisions made by others, but have little say in those decisions” despite their first‐hand knowledge about how policy affects patients and communities. They decry the under‐representation of nurses at the senior board level of major global health organizations.

The integration of genomics across health care provides nurses with leadership opportunities. In its report on the future of genetics and genomics in nursing and midwifery, the Task and Finish Group (2011, p. 10) stated that nurses could “accept the changes brought to healthcare by genomics as passive agents or they can be proactive in shaping and informing the transformation in their areas of practice.” We would argue that, through the complementary activities of the International Society of Nurses in Genetics (focusing on nurses in the specialist genomics field) and the G2NA (focusing on nurses outside the specialty), and others, nurses are being proactive and working to drive change. We also make the observation that the narrative in the nursing literature in this field is moving from “why” genomics in nursing to “how best” to integrate it. Nurses have already led internationally in establishing genomics competencies, by which the profession can increase its genomic translational capacity, a model since adopted by other healthcare professions (Calzone et al., 2018, 2018b). Williams et al. (2017) see nurses’ leadership roles in implementing genomics in precision health care as a key element for improved health. Calzone, Jenkins, Culp, and Badzek (2018) substantiated this following their rigorous, longitudinal study with designated nurse leaders to enhance nurses’ genomic competency in the hospital setting, using awareness campaigns and educational activities. They presented empirical evidence of the positive impact that nurse leaders play in facilitating integration, but concluded that more sustained investment in leadership education, infrastructure, and policy development was needed to embed change.

### Theoretical Framework

The challenge of implementing genomics, a complex and dynamic science, into health systems that are also complex, dynamic, and resource constrained, is one that Stark et al. (2019) described as “formidable” (p. 16), with the barriers to clinical implementation crossing many domains, driven in part by the need to share genomic data on a global scale and across diverse political, economic, and social contexts. Roberts, Kennedy, Chambers, and Khoury (2017) noted how the pace of genomic discovery outruns the pace of implementation and proposed that implementation science (IS; the study and application of methods and strategies that promote uptake of research findings and evidence into practice and policy) can help address these challenges. However, in their systematic review of 283 studies implementing genomic research, Roberts et al. (2017) found that few incorporated IS frameworks, sustainability measures, or capacity building, gaps they see as important.

Although there is a plethora of theoretical approaches within IS, Nilsen (2015) identified three common overarching aims of IS studies: describing or guiding the process of implementation; understanding what influences outcome; and evaluating the implementation. The Consolidated Framework for Implementation Research (CFIR) draws together 19 theories to provide a well‐accepted organizing framework for implementation programs, helping to identify what works (Damschroder et al., 2009). It comprises five domains: intervention, individuals, inner context, outer context, and process, each one underpinned by a number of constructs (39) believed to interact and influence implementation. Detailed descriptions are provided for each construct. The CFIR was adopted by the Implementing Genomics in Practice (IGNITE) Network to inform its approach to identifying relevant constructs and measures for evaluating implementation of genomics healthcare (Orlando et al., 2018). They identified 10 CFIR constructs as high priority. In nursing and genomics, behavior change theories have played an important role in implementation studies (Leach, Tonkin, Lancastle, & Kirk, 2016), with Diffusion of Innovations being perhaps the most widely used, such as in a large‐scale U.S. program (Jenkins et al., 2015). Williams et al. (2017) identified three key areas for nurses at the IS–genomics intersection (nurse leadership, genomics literacy, and learning from change) to understand the determinants of successful and failed implementation.

### Use of Roadmaps

Roadmaps can be used to provide a diagrammatic visualization of strategic plans to help organizations navigate from the present to the future. Although they are highly diverse in format, Blackwell, Phaal, Eppler, and Crilly (2008) found that one common benefit of the process of roadmap development is that it “brings together the various key stakeholders and perspectives needed to develop understanding of complex systems and issues, building consensus about the way forward” (p. 129).

Roadmaps have been widely used in healthcare planning, including by the WHO for large‐scale projects such as the Palliative Care Roadmap for countries with resource constraints (Callaway, Connor, & Foley, 2018). In the United States, the Centers for Disease Control and Prevention’s (CDC’s) National Public Health Workforce Strategic Roadmap details the strategies needed to reach four goals, through strengthening the public health and healthcare workforce to improve the public’s health (CDC, 2013). Manolio et al. (2013), on behalf of the U.S. National Human Genome Research Institute, convened 20 groups to discuss opportunities and challenges faced in implementing genomic medicine. They produced an implementation roadmap as a flow diagram to capture the approach at one institution. Here, we present what we believe to be the first roadmap for the strategic integration of genomics across nursing.

## Methods

### Aims

The aim of the initial G2NA program of work was to gather ideas and reach consensus on the core components of a roadmap to guide nurse leaders in promoting and monitoring incorporation of genomics across nursing professional practice at local and national levels.

The project was reviewed and approved by the Faculty of Life Sciences and Education Ethics Committee, University of South Wales (November 21, 2016).

### Participants

A purposive sampling strategy was adopted to recruit experts in nursing, policy, health care, and services in senior leadership roles, with as wide a geographical spread as possible. Expertise in genomics was not a prerequisite (Calzone et al., 2018, 2018a).

### Intervention

The study design combined online surveys with an interactive residential 3‐day workshop (at the Wellcome Genome Campus, Cambridge, UK). Data were gathered in three phases: prior to, during, and following the workshop (Table S1). A graphic facilitator was employed to capture a visual record and recapitulate discussion each day. Although mixed methods were adopted, the core approach to roadmap development was interactive and participatory, adopting the Liberating Structures approach “specifically designed to include, engage, and unleash everyone in contributing ideas and shaping their future” (Lipmanowicz & McCandless, 2013, p. 21). Eight activities from the 33‐item Liberating Structures menu were selected as most appropriate to the purpose of specific activities within the workshop (see Table S1). Participants worked in pairs, in small groups, or as a single (large) group. Activities were facilitated by the investigators, and electronic voting was used to provide instant (anonymous) feedback or to assess consensus on points of discussion. A maturity matrix to guide and benchmark professional and organizational progress in genomics integration was developed using iterative participatory work to agree with the critical success factors (CSFs) and subcomponents of a tool subsequently refined and piloted amongst the participants (Tonkin et al., submitted).

Following the workshop and cognizant of all of the components of the maturity matrix and workshop outputs, a group was convened from within the steering group (composed of the investigators) and those who volunteered (*n* = 12) at the workshop to join the strategic planning writing or reading groups. The aim of the writing group was to articulate the mission, vision, and strategic objectives and actions of the G2NA strategy. The reading group was asked to provide detailed, free‐text feedback on all aspects of the plan, with any changes or recommendations for additional strategies or collaborative partnerships, addressed in the final draft.

The conceptual framework underpinning the roadmap development and its complementarity with the maturity matrix and G2NA strategy is depicted in Figure S1. The roadmap was informed by all outputs collected over the two phases of the project. It also draws on the Consolidated Framework for Implementation Research (Damschroder et al., 2009) to inform the process for implementation of the roadmap and for pre‐implementation assessment.

### Analysis

Where relevant, data were exported to Excel. Analysis was conducted using descriptive statistics and thematic analysis of items generated, using two coders from the project team.

## Findings

The results from the pre‐workshop surveys have been published previously (Calzone et al., 2018a, 2018b); they are summarized here for completeness, to provide the context for the components of the roadmap development. Participants from 19 countries in total were involved; 18 responded to the survey, and of those, two were unable to attend the workshop. However, a participant from an additional country was able to attend at short notice, after the survey had closed. In addition to the six project team members, there were 23 participants at the workshop, with representatives from 17 countries and seven organizations (for full details see Tonkin et al., submitted).

### Genomics Services and Nursing

The global landscape survey of nursing and genomics revealed that regulation, training, qualifications needed, and the nurses’ role generally was varied, with the low status of nursing seen as a priority area for action (Calzone et al., 2018, 2018a). Although genetics services were provided in all 18 countries, provision was diverse, from one country where newborn screening only was offered, to those few where genomics services for common diseases were widely available outside specialist centers. Only five countries recognized the specialist genetics nurse role with the role varying between countries. Four of the countries had independently defined training standards for specialist genetic nursing. Although this study focuses on nongenomics nurses (i.e., the majority), the findings in relation to genomics are pertinent. In their survey of 10 countries, Kirk, Calzone, Arimori, and Tonkin (2011) found that those more advanced in genomics integration across nursing shared two critical factors: the status of genetics services and the formal recognition and regulation of the specialist genetics nurse role. They thus may act as an indicator of the profession’s broader preparedness.

### Resources

Participants were asked about resources at organizational and country levels under the five broad categories shown (Table S2). Those participants identifying education resources were asked to detail their nature, scope, and accessibility, including IT infrastructure to facilitate learning and networking (for full details see Calzone et al., 2018, 2018b). Although a range of genomics resources were identified, these were associated with only nine countries at most (education resources), and were sparse in the other four categories. Particular gaps were noted around awareness and accessibility of available resources and availability of nursing‐focused resources. Workshop participants agreed that a global effort to curate, share, and develop resources, particularly those that target nurses, should be a priority for action.

### Barriers, Facilitators, and Actions Needed

The survey revealed broad consensus on the barriers to integration of genomics into nursing practice and education (Calzone et al., 2018, 2018a), and these were reiterated during the workshop. Limited availability and access to education, resources, and expertise were seen as problematic, and particularly for education, the lack of leadership and agreed guidelines on training or standards was highlighted. Unsurprisingly, the priority areas for action reflected the focus on education, but also highlighted the professional status of nursing, in genomics and more widely. Following the workshop, the barriers and priorities identified were mapped to 24 of the 31 constructs across four domains of the CFIR (Damschroder et al., 2009; Figure S2a), indicating the relevance of the framework to this study.

When asked for ideas on who nurse leaders could approach for help on implementing genomics, 117 separate suggestions were made across a range of categories (Table S3). Nursing societies and genomics societies generated the most items (*n* = 22 each).

Participants nominated 22 ideas about how best to promote genomics integration. The eight scored the highest by peers can be categorized broadly as those around the formal establishment of the G2NA, developing a global community of practice to share resources and mentor others, raising awareness about genomics and the nursing role to influence practice, and influencing policy at the strategic level.

### Maturity Matrix

The CSFs for effective family‐ and community‐focused nursing care that result in improved health outcomes through incorporation of genomics were agreed as:

- Enhanced education and workforce development;
- Effective nursing practice that builds on an evidence base, with clear delineation of nursing roles and interventions in delivering genomic health care;
- Infrastructure and resources to support incorporation of genomics in education, practice, and services;
- Collaboration and communication on a shared vision of genomic health care across borders and professional groups;
- Person‐, family‐, and community‐focused care, with accessible services and an informed public;
- Leadership in transforming health care through policy development.

The 19 key enablers identified across the CSFs (Table S4), along with indicators, progress benchmarks, and measures to evidence progress along a predefined trajectory, were synthesized to form the Assessment of Strategic Integration of Genomics across Nursing (ASIGN) planning and assessment tool for nurse leaders (Tonkin et al., submitted). Flexibility to local context is accommodated through progress being measured against stage of change of maturity (from pre‐contemplation to leading, over five stages) rather than a timeline, with assessment using locally appropriate measures. This is an important factor in supporting a globally applicable roadmap.

We intend that ASIGN would be used as a planning instrument to benchmark the current status of genomics integration through local self‐assessment, and then guide planning and activities to drive progress to the next stage of maturity along the ASIGN matrix. An iterative process that also incorporates elements from the roadmap planning and evaluation is adopted to assess change for each stage of maturity. However, a larger pilot and longitudinal studies are needed to gauge effectiveness of ASIGN.

### G2NA Strategic Planning to Inform and Support the Roadmap

The goals encapsulated in five of the CSFs were organized into three core strands: advocacy, evidence‐based practice, and collaboration and public engagement. A cross‐cutting foundational strand of sustainable infrastructure and resources was also articulated. Six G2NA strategic objectives were agreed upon (Figure S3), with underpinning strategies and actions. These provide a framework of practical steps that the G2NA can take to support each CSF, enabling us to fulfil our mission and to realize our vision of the G2NA acting as the unified international voice for advancing and integrating genomics across the nursing practice, supporting nurse leaders and facilitators implementing the roadmap.

Accelerating genomics discovery and implementation, sharing best practice, collaboration, and education are common strands to be found in many large‐scale genomics medicine programs such as the U.K. 100,000 Genomes Project (Turnbull et al., 2018) and the U.S. National Human Genome Research Institute–funded eMERGE Network (Gottesman et al., 2013). The themes captured by the maturity matrix CSFs, the G2NA vision and mission, strategic objectives, and overarching themes of advocacy, evidence‐based practice, and collaboration and public engagement echo these strands. This is perhaps unsurprising given that they share a common goal, ultimately of improving health through genomics. Perhaps the closest to the G2NA goals and mission is the Global Genomics Medicine Collaborative (G2MC; Ginsburg, 2019). Its key platforms are education and workforce, policy, and advocacy, with collaboration as a key theme. The focus is on medical clinicians and scientists, with nursing having a low profile but the G2MC, like the G2NA, sees the value in interprofessional collaboration. However, we believe the G2NA is the only global genomics initiative specifically targeting all nurses, irrespective of specialty.

### The Roadmap

**Figure 1:**
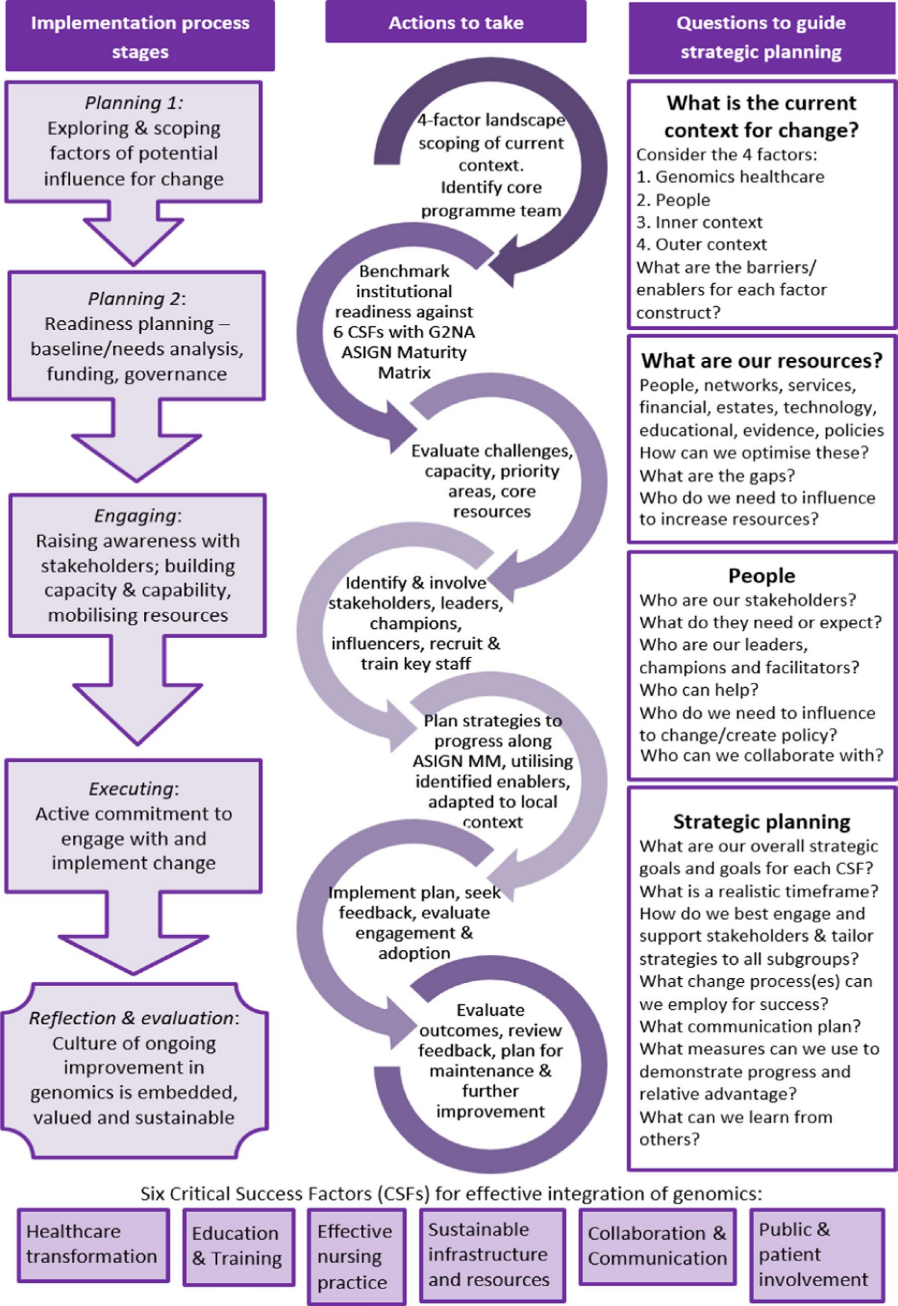
Global Genomics Nursing Alliance Roadmap for nurse leaders to implement genomics across nursing. [Colour figure can be viewed at wileyonlinelibrary.com]

With the information on the core components now gathered, the G2NA Roadmap could be articulated. While ASIGN is the key instrument for the roadmap application, we wanted to set it into a practical context as guidance for those nurse leaders who themselves already have sufficient awareness and understanding of genomics to appreciate its relevance to health care and the need for coordinated strategic action, even though they might not themselves be expert. The strategic goals of the G2NA indicate the scale of the challenge, with the focus on leadership, policy change, and education. The need to support arguments with an evidence base is also acknowledged, along with collaboration and communication. Public engagement ensures that patients and families remain at the core of activity. Complex, large‐scale change has to be managed strategically and systematically, and the CFIR framework of Damschroder et al. (2009) offers a comprehensive approach to achieving this. Many of the issues raised during the course of this study are not necessarily unique to genomics, but they do offer a perspective for nurses on the CFIR constructs through the genomics lens. The roadmap depicted (Figure 1) follows the CFIR implementation process stages: planning; engaging (including influencers, champions and leaders); executing; and reflecting and evaluating (see Figure 1, left hand column). Evaluation is also built in throughout the process. Actions to be taken as an expansion of each stage, in tandem with utilization of ASIGN, are shown in the center column. These take the user through one complete cycle of ASIGN self‐assessment, from initial benchmarking to the follow‐up progress assessment. The third column poses questions to consider as part of a systematic, pre‐implementation assessment to identify barriers, facilitators, and priorities, informed by CFIR constructs (see Figure S2).

## Discussion

We have presented the first roadmap to guide implementation of major change initiatives to promote the integration of genomics across nursing that is adaptable to local context and of global relevance. The need for change is acknowledged across the literature, as are the common challenges. We believe that a practically focused roadmap can help move the agenda forward by answering “what do we do next?” Our intention is to create a more interactive roadmap online where the user can navigate to more detailed information gathered in this study related to identified barriers and facilitators, available resources, and in response to the question “who can help?” We recognize it will need updating as new knowledge comes along, not just in genomics, but also as the evidence base for nursing practice builds and health systems evolve. However, we believe that the benchmarking dimension of the G2NA Roadmap (delivered via ASIGN) will provide an instrument to evaluate quality improvement initiatives at local and national levels, guide progress along a given trajectory, and provide evidence for allocation of resources.

Three interlinked essential elements emerged through this research program: resources, leadership, and collaboration. The need for accessible, relevant educational resources was a clear priority, but participants also raised issues around access to genomics experts, support networks, infrastructure, and leadership. This highlights the importance and relevance of the status and availability of nurses within the specialist genomics field to be able to act as an expert resource and for nurses from both within and outside the field to work together to advance genomics integration. Gilbert, von Ah, and Broome (2017) consider the interrelationship between human capital (knowledge and expertise of individuals), social capital (which can be broadly described as the resources inherent in networks and the people within them), and organizational capital (the organizational knowledge, culture, structures, processes, and policies within an organization). They propose a conceptual model to explain how the nurse leader has a direct moderating influence on both human and social capital within a healthcare organization. This resonates with the findings (and general discussion) throughout this project and echoes the work by Williams et al. (2017) and Calzone, Jenkins, et al. (2018) on the importance of nursing leadership in promoting genomics integration. Kitson and Harvey (2016) highlighted the role of the facilitator more generally in their work on effective implementation of new knowledge in clinical practice. They described the facilitator as the “active ingredient” and identified three levels of facilitator: novice, experienced, and expert. This is perhaps particularly relevant in genomics integration, where capacity is a challenge and expertise a limited commodity. Nurses new to genomics but eager to learn may be targeted for training to act as ambassadors or champions for genomics integration.

Stark et al. (2019) see a collaborative approach as essential to successful implementation of genomics, with global alliances and networks helping to create “a global learning healthcare system to enable rapid translation” (p. 18). The success of the G2NA program and its associated activities was dependent on the willingness of individuals to accept and understand different perspectives, collaborate, and share expertise and resources, and is foundational to the establishment of the G2NA itself. The benefit of the roadmap development process articulated by Blackwell et al. (2008) was realized as a result of the process we followed, bringing unanimous consensus on the way forward and a strong endorsement of the approach used.

### How Should the Roadmap Be Used Next?

Our intention is that the G2NA Roadmap should be of global relevance, adaptable to local contexts, with the common goal of genomic integration to improve healthcare for all. Promoting inclusivity and equity is an important aspect of this. Alcaraz et al. (2017) introduced their ConNECT framework as a model for fostering health equity in the behavioral sciences, which Menon, Cohn, Downs, Gephart, and Redwine (2019) also see as applicable to integration of precision medicine. The model espouses five broad and synergistic principles. The first principle advocates that integration of context (in this case genomics) should also place emphasis on understanding the broader social and contextual influences. The second principle advocates for inclusivity, particularly for marginalized, minority, and vulnerable populations. Complementary to this, the third principle focuses on ensuring accessible diffusion of innovations, and understanding the barriers and facilitators for this. Gaining stakeholder perspectives embeds communication as a dialog and further supports inclusivity. The fourth principle focuses on effective utilization of communication technologies to promote efficient sharing of information, inclusivity, and health equity. The fifth principle emphasizes the need to promote specialized training and continuing education that also encompasses cultural and linguistic competence. This set of broad guidelines can be used by teams to consider how their implementation plans address the issues of equity and inclusivity. We encourage their application. The roadmap sets the maturity matrix into a well‐tested IS framework (CFIR; Damschroder et al., 2009), further informed by ConNECT principles (Alcaraz et al., 2017), to guide planning and implementation for nurse leaders to drive the integration of genomics across nursing at local and global levels. It thus offers a unique opportunity to apply IS methodologies to a large‐scale genomics initiative specifically targeting nursing.

## Limitations

Funding constraints dictated that the number of participants be small, and representation from more countries and organizations was not feasible. Moreover, the purposive sample was selected to include individuals outside of the genomics specialty to capture perspectives from national and global policymakers, education, practice, research, and the patient voice, as well as insights from individual countries. Each brought expertise from their specialist area but may not have had full knowledge of other areas. However, the methods used throughout the workshop aimed to promote common understanding. Nonetheless, we cannot claim that the views shared were representative of a wider population of experts, and the findings should be considered in that context. However, they do provide a foundation on which to build.

## Conclusions

The large‐scale change needed throughout health systems globally to integrate genomics is indeed formidable. Our work has shown that agreement can be reached among experts from diverse cultures and backgrounds on the scope and nature of effort needed to address this. Willingness to advance it through a collaborative effort was also evident. The creation of the G2NA Roadmap for global acceleration of genomics across nursing is another step forward. It places strategic nursing leadership at the heart of the ultimate goal of the G2NA, to improve health for all, through realizing the potential of all nurses in delivering genomics health care. We believe it is the first global organization to do so.

The words of one participant (a patient advocate) in voicing a “big idea” are pertinent here: “To see a world where patients and families in need of timely expert advice and support from nurses properly trained and supported in genomics, are able to receive it without undue delay.”

## Supporting information


**Figure S1.** Conceptual framework and context for building the Roadmap.
**Figure S2.** Using the Consolidated Framework for Implementation of Research (CFIR) (Damschroder et al., 2009) as an organising framework for genomics integration across nursing.
**Figure S3.** Diagram to show the G2NA strategic objectives underpinning the Critical Success Factors to fulfilling the G2NA mission.
**Table S1.** Methods Used Through the Stages of Roadmap Development, With Outcomes.
**Table S2.** Summary of Survey Findings on Genetics Resources for Nurses (adapted from Calzone et al., 2018b).
**Table S3.** Summary of Facilitators Identified, by Category: Who Can Help?.
**Table S4.** The Six Critical Success Factors (A‐F) With Key Enablers for Effective Integration of Genomics Across Nursing Care to Promote Improved Health Outcomes (adapted from Tonkin et al., submitted).
