# Supplementary material for: A Roadmap for Global Acceleration of Genomics Integration Across Nursing
Source: J Nurs Scholarsh. 2020 Apr 17;52(3):329–38. doi: 10.1111/jnu.12552 (PMC7202994; doi:10.1111/jnu.12552)
Supplement: Supplementary file 1 — Figure S1. Conceptual framework and context for building the Roadmap. Figure S2. Using the Consolidated Framework for Implementation of Research (CFIR) (Damschroder et al., 2009) as an organising framework for genomics integration across nursing. Figure S3. Diagram to show the G2NA strategic objectives underpinning the Critical Success Factors to fulfilling the G2NA mission. Table S1. Methods Used Through the Stages of Roadmap Development, With Outcomes. Table S2. Summary of Survey Findings on Genetics Resources for Nurses (adapted from Calzone et al., 2018b). Table S3. Summary of Facilitators Identified, by Category: Who Can Help?. Table S4. The Six Critical Success Factors (A‐F) With Key Enablers for Effective Integration of Genomics Across Nursing Care to Promote Improved Health Outcomes (adapted from Tonkin et al., submitted). [file JNU-52-329-s001.docx]

Phase 1: Pre-workshop and workshop activities

**Developing a tool for Assessment of Strategic Integration of Genomics across Nursing (ASIGN)**

Using a Maturity Matrix to guide & benchmark effective integration of genomics across nursing

For each ASIGN CSF, identifying key enablers; indicators; benchmarks for each stage of maturity

Phase 2: Post-workshop dialogue, planning and refinement

**G2NA Mission:**

**Improving healthcare for all through supporting the nursing professions to integrate genomics across nursing education and practice**

**G2NA Roadmap**

Using CFIR^1^ to set ASIGN and other outcomes in a practical context for Nurse Leaders seeking to promote integration of genomics across nursing

Phase 1 outcomes

Refining ASIGN and pilot testing

**G2NA Strategic Planning**

Articulating the G2NA Vision, Mission, Strategic Goals & Priorities for action

^1^ Consolidated Framework for Implementation Research (Damschroder et al., 2009)

**Figure S1.** Conceptual framework and context for building the Roadmap.

**a.** The CFIR constructs and sub-constructs across the four domains of particular relevance to genomics integration across nursing. Potential barriers and priority areas (bold) identified in this study through survey, workshop activities and strategic planning.

**1. INTERVENTION**: Intervention source, **Evidence strength and quality**, Relative advantage, **Adaptability**, Trialability, Complexity, Design quality & packaging (**Accessibility**), **Cost.**

**2. PEOPLE**: **Knowledge & beliefs about genomics**, **Self-efficacy**, **Individual** **Stage of Change**, Individual identification with organisation, Other personal attributes.

**3. INNER CONTEXT:** Structural characteristics, **Networks & communications**, **Culture**, **Implementation climate**: **Tension for change**; **Compatibility**; **Relative Priority**; **Organisational incentives and rewards;** Goals and feedback; **Learning climate**, **Readiness for implementation**: **Leadership engagement**; **Available resources**; **Access to knowledge and information.**

**4. OUTER CONTEXT**: **Patient needs & resources**, **Cosmopolitanism**, **Peer pressure**, **External policy and incentives.**

**b.** Using the domains and associated constructs as a basis for 4-factor landscape scoping to guide Roadmap implementation planning

For each construct in a CFIR domain, asking the following questions:

N.B. A full list of CFIR constructs with short definitions may be found in Damschroder et al. (2009) Additional file 3 [http://www.biomedcentral.com/content/supplementary/1748-5908-4-50-S3.pdf].

**Figure S2.** Using the Consolidated Framework for Implementation of Research (CFIR) (Damschroder et al., 2009) as an organising framework for genomics integration across nursing.

**Table S1.** Methods Used Through the Stages of Roadmap Development, With Outcomes

| **Method** | **Purpose** | **Outcomes** |
| --- | --- | --- |
| Pre-workshop | |  |
| Webinars; online briefing packs | Preparatory briefing of participants to gain insight into project aims and wider context | Establishing a common platform |
| Online survey | Landscape analysis genomics & nursing | Paper published |
| Online survey | Genomics resources survey | Paper published |
| Email | Ideas generation for critical success factors to genomics integration in nursing | 63 items, categorized under 7 themes. |
| Poster preparation | Each participant to set the context for healthcare, nursing and genomics for their country/organisation, using a fixed template | Gaining understanding of wider context; Online poster booklet |
| Workshop day 1 | |  |
| Impromptu networking^1^ | Share challenges, expectations and build connections | Task completed. |
| Conversation Café with poster review^1^ | Shared understanding of complex situations, generate new ideas; review current resources | Common challenges and goals identified. |
| What i need from you^1^ | Gather insight and priorities from each participant; Share context and needs, prioritise | Task completed. Common ground and needs identified. |
| Interactive presentation with electronic voting | Achieve consensus on Maturity Matrix Domains/ Critical Success Factors (CSFs) from 7 themes generated pre-workshop | 7 themes merged into 6 Critical Success Factors, by consensus |
| Workshop day 2 | |  |
| Group discussion | Graphic art review to capture highlights and perspectives (Day 3 also) | Series of graphic art posters. |
| Shift and share^1^ | Small group iterative process to identify core components of each CSF of the Maturity Matrix | Framework and core components of the Maturity Matrix agreed. |
| Panarchy^1^ | Group review to identify stages of maturity and Maturity Matrix outcome measures |  |
| Workshop day 3 | |  |
| 25/10 Crowd sourcing^1^ | Generate and sift the group’s most powerful and actionable ideas | 22 ideas nominated. |
| 15% Solutions^1^ | Identify ‘what is possible now’, identify relevant organisations ‘who can help’ | See Table S3 |
| Wise Crowds^1^ | Discussion with voting to identify gaps, areas for further refinement and priorities | Priorities agreed, key gaps identified. |
| Post workshop | |  |
| Maturity Matrix refinement: steering group discussion  online survey | Further refinement and pilot testing of ASIGN (Assessment of Strategic Integration of Genomics across Nursing) tool | Pilot testing completed Paper submitted, undergoing revision. |
| G2NA Strategic planning: discussion and review (e-mail/ face to face/WebEx) | Establish writing group (6, including 3 volunteers) and reading group (9 volunteers). Articulate vision, mission, strategic objectives and actions. Feedback on Strategic Plan | Strategic plan drafted, revised following feedback. Final plan completed. |
| Roadmap development: discussion and review | Collate outputs and review context. Set ASIGN into implementation framework | Roadmap completed, to undergo wider testing with ASIGN. |

^1^Methods adopted from Liberating Structures (http://www.liberatingstrucures.com)

**Table S2.** Summary of Survey Findings on Genetics Resources for Nurses

(adapted from Calzone et al., 2018b)

| **Resource** | **Comments** |
| --- | --- |
| Genetics competency frameworks/curriculum guidelines for nurses | Available in 3 countries and Europe |
| National organisations for genetic nurses and/or counsellors | Available in 5 countries, with one international organisation (International Society of Nurses in Genetics) |
| Certification for genetic nurses and/or counsellors | Required in 4 countries |
| Visible nursing leadership in genomics at national level | 6 participants reported visible leadership in their country |
| Education resources | 9/23 participants identified resources in their own country. Courses in genetics/genomics were reported in 6 countries. Web-based resources (30 items) were reported from 8 countries. |

NB Resources indicated in this table and in Table S3 are those identified by participants. We recognise that there will be omissions, such as national credentialing bodies which may be of relevance. These should be scoped at national level when individuals/organisations look to implement the roadmap.

**Table S3.** Summary of Facilitators Identified, by Category: Who Can Help?

| **Category** | **Number of items** | **Comments** |
| --- | --- | --- |
| Nursing societies | 22 | 10 national, 1 regional, 4 international, includes ICN (major role in global health policy). 7 specialist nursing societies including 3 cancer nursing groups. |
| Genetics societies | 22 | 13 national, 4 regional, 5 international. Includes Genomic Nursing Expert Panel sub-group of American Nurses Association. |
| Genetics nursing/ counselling societies | 12 | 9 national, 1 regional and 2 international societies. |
| Patient support organisations | 11 | 7 national, 2 regional, 2 international. 5 with specific Rare Disease focus. |
| Academic bodies | 10 | University-based, with focus on education and research |
| National government bodies and departments | 9 | Includes National Institutes for Health and relevant departments, Health Education England, and Punjab Thalassaemia Prevention Programme |
| World Health Organisation | 8 | WHO and 7 relevant programmes/ WHO Collaborating Centres |
| National nurse educators leadership groups | 5 | Deans and heads of schools of nursing |
| National nursing regulatory bodies | 5 | Focus on standards and policy |
| National nursing professional organisations | 3 | Includes UK Royal College of Nursing |
| Other | 6 | 2 independent policy advisory organisations, 3 charities, 1 communications company |
| Other medical/professional organisations | 4 |  |
| **Total** | **117** |  |

**Table S4.** The Six Critical Success Factors (A-F) With Key Enablers for Effective Integration of Genomics Across Nursing Care to Promote Improved Health Outcomes (adapted from Tonkin et al., submitted)

| **Critical success factor** | **Key enablers** |
| --- | --- |
| A. Enhanced education and workforce development | A1 Culture of positive attitudes towards nursing and genomics at all levels, that is locally sensitive and builds on wider public awareness.  A2 Core competencies in genomics which are culturally sensitive, responsive and engender critical analytic skills; realistic at the macro level taking into account the existing nursing workforce, across all levels, roles, and clinical specialties.  A3 Trained faculty, including adjunct staff and clinical preceptors, who are confident, competent and collaborative in genomics. |
| B. Effective nursing practice that is person/family-centred, evidence-informed, safe, ethical and clearly defined | B1 Evidence informed practice to enhance quality care.  B2 Clearly defined role of the practising nurse in applying genomics in care delivery.  B3 Clearly defined patient outcomes related to genomic care.  B4 Ethical and safe practice utilizing genomics. |
| C. Sustainable infrastructure and resources that support incorporation of genomics into education and practice | C1 Service capacity  Genomics clinical and testing services are delivered to agreed and accredited standards, underpinned by sustainable infrastructure that includes human resources.  C2 Political and financial investment to support adequate and appropriate workforce development and sustainable genomics service delivery, with commitment at strategic policy levels.  C3 Human Resources that support nursing career potential: within the genomics sub-specialty; and for all nurses who incorporate genomics. |
| D. Collaboration and communication to create social capital to accelerate change | D1 Strong working relationships which are collaborative, may be multidisciplinary, and which result in positive outcomes, influence, impact and advocacy.  D2 Collaboration across boundaries to share genomics knowledge, expertise and resources to facilitate education and practice.  D3 Effective communication across stakeholder groups. |
| E. Public and patient involvement that is core to policy and practice | E1 Commitment to enhance health literacy in genomics for the public, society and healthcare services through engagement and dialogue.  E2 Access to services.  E3 Patient involvement and empowerment (and family as part of the health care system). |
| F. Healthcare transformed through policy and leadership | F1 Shared vision of the essential contribution of nurses to improved health outcomes through delivering genomic healthcare.  F2 Drive within the profession from nurse leaders to realise the vision through creating and influencing policy.  F3 Engagement of policy-makers (macro to micro levels) in support for the nursing role in delivering genomic healthcare. |


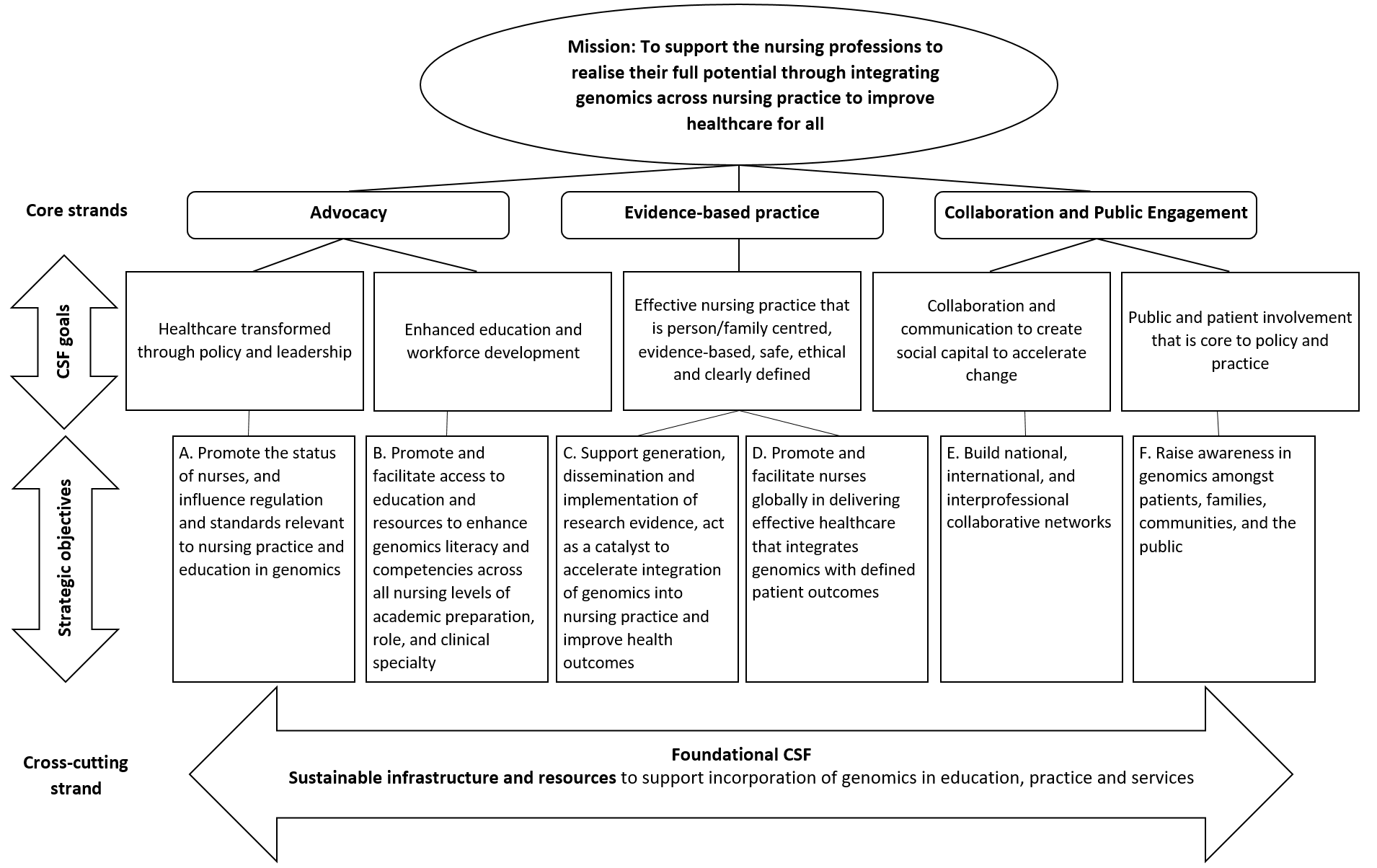


**Figure S3.** Diagram to show the G2NA strategic objectives underpinning the Critical Success Factors to fulfilling the G2NA mission.
